# Rural versus urban pediatric non-accidental trauma: different patients, similar outcomes

**DOI:** 10.1186/s13104-018-3639-4

**Published:** 2018-07-28

**Authors:** Ashley P. Marek, Rachel M. Nygaard, Ellie M. Cohen, Stephanie F. Polites, Anne-Marie E. Sirany, Sarah E. Wildenberg, Terri A. Elsbernd, Sherrie Murphy, D. Dean Potter, Martin D. Zielinski, Chad J. Richardson

**Affiliations:** 1 Department of Surgery, Hennepin County Medical Center, 701 Park Ave S, P5, Minneapolis, MN 55415 USA; 2 Department of Surgery, Mayo Clinic, 200 First Street SW, Rochester, MN 55905 USA

**Keywords:** Non-accidental trauma, Intentional trauma, Pediatric trauma, Pediatric injury, Urban injury, Rural injury

## Abstract

**Objective:**

Our aim was to compare urban and rural non-accidental trauma for trends and characterize where injury prevention efforts can be focused. Pediatric trauma patients (age 0–14 years) at two level I adult and pediatric trauma centers, one rural and one urban, were included and data from the trauma registries at each center was abstracted.

**Results:**

Of 857 pediatric admissions, 10% of injuries were considered non-accidental. The mean age for all non-accidental trauma patients was significantly lower than the overall pediatric trauma population (2.6 vs. 7.7 years, P < 0.001). Significantly more fatalities occurred in the non-accidental trauma cohort (5.7% vs. 1% P = 0.007). In nearly half of all non-accidental trauma patients, the primary insurance was government programs (49%) and 46% were commercial insurance. The proportion of government insurance in non-accidental trauma was higher in both urban and rural cohorts. There were similar rates of urban and rural patients sustaining non-accidental trauma who were uninsured (6.5 vs. 5.3%). Patients that were younger, in a rural location, and receiving government insurance were at higher risk of non-accidental trauma on univariable analysis. However, only age remained an independent predictor on multivariable analysis.

## Introduction

Injury is the most common cause of morbidity and mortality in children within the United States. Long-term complications, including depression [1], chronic pain [2], and physical functional deficits that include difficulty with mobility and self-care [2–4], have been seen in almost half [3] of injured children. Victims have also been shown to have a quality of life deficit as compared to their peers [2, 5].

Types of injuries and outcomes after pediatric trauma vary based on age, sex, income, and area of residence [6]. Specifically, studies comparing rural and urban pediatric trauma generally demonstrate higher incidence and mortality in rural areas [6–8], while data from Canada [9] suggests non-accidental trauma is more prevalent in urban areas. Non-accidental trauma in the United States is associated with more severe injury and worse outcomes than unintentional injuries [4, 8, 10].

Despite existing data demonstrating the impact of environmental circumstances on pediatric trauma, little is known about the difference in epidemiology and outcomes after injury in children treated at urban versus rural pediatric trauma centers. This information would be useful in informing tailored injury prevention efforts and in ensuring pediatric trauma centers are prepared to care for their endemic populations. Our aim was to compare patterns of urban and rural non-accidental trauma, to evaluate for potential trends and identify cases where injury prevention efforts can be focused. Identifying factors associated with non-accidental trauma may function as a cue for providers to conduct further assessment in cases of pediatric trauma potentially preventing future harm. We hypothesized higher mortality in non-accidental compared to accidental trauma and higher severity of injury in urban non-accidental trauma compared to their rural cohort.

## Main text

### Methods

To test our hypothesis, we gathered data from two American College of Surgeons level I adult and pediatric trauma centers, one in an urban setting and one in a rural area of the same state. All pediatric trauma admissions from May 2010 through July 2011 were identified in the trauma registries at the two centers. Trauma registries are maintained by trained abstractors according to guidelines set forth by the American College of Surgeons. Pediatric patients were defined as being between 0 and 14 years old at the time of admission. Patients with burn injury were excluded from the analysis, as only one of the hospitals includes an American Burn Association-verified burn center. Data collected included treatment center (urban or rural), patient age, gender, mechanism of injury (blunt or penetrating), injury severity score (ISS), hospital length of stay, outcome, and payer group. Intent of injury was recorded in the registry based on the Centers for Disease Control matrix of E-code groupings for injury as non-accidental, accidental, self-inflicted, undetermined, or other. We included only patients with intent coded as accidental or non-accidental in our study.

The Institutional Review Board for Human Subject Research Committee approved the study at each institution in accordance with the ethical standards of the institution and with the Helsinki declaration. This research involved retrospective data collection, therefore formal consent is not required for this type of study and all identifying information was eliminated following completion of data gathering. We first compared non-accidental and accidental trauma in the entire cohort, followed by a comparison of non-accident trauma in the urban and rural cohorts. To test for similarities and differences we conducted the Chi^2^ test for categorical variables, Student’s *T* test for comparison of means, and Mann–Whitney test for comparison of medians. We also conducted regression analysis to identify potential factors associated with non-accidental trauma while controlling for potential confounding variables. Univariable and multivariable logistic regression was conducted to assess factors associated with non-accidental trauma. Statistical significance was set for two-sided tests at P ≤ 0.05. Statistical analysis was conducted using STATA 14.1 (College Station, TX).

## Results

Of 857 patients, the majority were treated at the rural center (54% vs. 46%). There were no significant differences between the urban and rural patients with regard to age (mean 7.2, SD 4.4), sex (65% male), ISS (median 4), or mortality (1.5%). Length of hospital stay (3.8 vs. 2.7, P = 0.01) and penetrating injury (9% vs. 5%, P = 0.024) were significantly higher in the urban cohort. The most common mechanism of injury for both groups was fall (35%). Rural children were less frequently transported by ambulance than urban children (41.6% vs. 64.2%, P < 0.001). Children treated at the rural center were more likely to be uninsured compared to urban children (4% vs. 7%, P < 0.001), while urban children were more frequently covered by government insurance (43% vs. 26%, P < 0.001). We found no significant difference in mortality between urban (2%) and rural (1.1%) children (P = 0.274).

Overall, 10% of injuries were considered non-accidental (Table 1). The mean age for all non-accidental trauma patients was significantly lower than the overall pediatric trauma population (2.6 vs. 7.7, P < 0.001). Significantly more children treated at the rural center were impacted by non-accidental trauma compared to the urban center (12.3% vs. 7.9%). Additionally, significantly more fatalities occurred in the non-accidental trauma cohort (5.7% vs. 1%, P = 0.001). A higher proportion of children with government insurance were impacted by non-accidental trauma (Table 1).

**Table 1:** Comparison of accidental and non-accidental trauma in the upper Midwest

	Accidental trauma (N = 769)	Non-accidental trauma (N = 88)	P value^a^
Age (y), mean (SD)	7.7 (4.2)	2.6 (3.2)	< 0.001
Male, N (%)	510 (66.3)	50 (56.8)	0.076
ISS, median (CI)	4 (4, 4)	9 (9, 9)	< 0.001
LOS days, mean (SD)	3.0 (5.6)	4.8 (9.8)	0.011
Mortality, N (%)	8 (1.0)	5 (5.7)	0.001
Mechanism of injury blunt, N (%)	706 (92.5)	87 (98.9)	0.026
Urban versus rural, N (%)			0.037
Urban	361 (92.1)	31 (7.9)	
Rural	408 (87.7)	57 (12.3)	
Payer group, N (%)			0.010
Government	246 (32.3)	43 (48.9)	
Commercial	447 (62.4)	40 (45.5)	
Uninsured	41 (5.4)	5 (5.7)	

*ISS* injury severity score, *LOS* length of (hospital) stay, *y* years

^a^Chi^2^ test for categorical variables, Student’s *T* test for comparison of means and Mann–Whitney test for comparison of medians

Specifically examining children impacted by non-accidental trauma, there was no significant difference in age, gender, or mortality between the patients treated at the rural or urban center. (Table 2). A larger proportion of non-accidental trauma patients were insured by governmental programs in both the urban (71% vs. 43.4%) and rural cohorts (36.8% vs. 26%). Patients that were younger, at the rural center, and receiving government insurance were at higher risk of non-accidental trauma on univariable analysis (Table 3). However, only age remained an independent predictor on multivariable analysis (Table 3).

**Table 2:** Characteristics of non-accidental trauma

	Urban (N = 31)	Rural (N = 57)	P value^a^
Age (y), mean (SD)	2.2 (2.8)	2.8 (3.4)	0.418
Male, N (%)	20 (64.5)	30 (52.6)	0.282
ISS, median (CI)	9 (5, 9)	9 (9, 9)	0.539
Mortality, N (%)	2 (6.5)	3 (5.3)	0.818
LOS days, N (%)	6.2 (12.2)	4.0 (8.3)	0.323
Mechanism of injury blunt, N (%)	31 (100)	56 (98.3)	0.458
Payer group, N (%)			0.006
Government	22 (71.0)	21 (36.8)	
Commercial	7 (22.6)	33 (58.9)	
Uninsured	2 (6.5)	3 (5.3)	

*ISS* injury severity score, *LOS* length of (hospital) stay, *y* years

^a^Chi^2^ test for categorical variables, Student’s *T* test for comparison of means and Mann–Whitney test for comparison of medians

**Table 3:** Regression analysis of factors associated with non-accidental trauma in the upper Midwest

	OR (CI)	P value^a^	OR (CI)	P value^b^
Age	0.66 (0.59, 0.72)	< 0.001	0.67 (0.60, 0.73)	< 0.001
Sex male	0.69 (0.47, 1.05)	0.078	–	
Rural	1.63 (1.03, 2.58)	0.038	1.55 (0.93, 2.59)	0.094
Mechanism of injury penetrating, N (%)	0.14 (0.02, 1.04)	0.055	–	
Payer group, N (%)				
Government	2.08 (1.32, 3.29)	0.002	1.37 (0.82, 2.29)	0.230
Commercial	Ref		Ref	
Uninsured	1.45 (0.54, 3.89)	0.455	1.08 (0.37, 3.14)	0.885

^a^Univariable logistic regression

^b^Multivariable logistic regression

## Discussion

Trauma remains the leading cause of death among children and adolescents in the United States. In Minnesota, the unintentional injury death rate from 2001 to 2005 was 13.6 per 100,000, which is lower than the national average of 15 per 100,000 [12]. We found that victims of non-accidental trauma had an even higher mortality than victims of unintentional trauma which is similar to other studies comparing the two [11–13]. However, we reject part of our hypothesis; we found no significant differences in injury severity in urban versus rural non-accidental trauma. Only age remained a significant predictor of non-accidental trauma following adjustment for confounding variables.

In looking specifically at differences in the rural and urban population, our study cohort contained a higher percentage of patients treated at the rural center. While there were differences in mechanism and payer group, there were no differences in injury severity or mortality when comparing centers. This differs from previous studies that have consistently demonstrated a higher risk of overall injury [6, 13–15] and higher rates of fatal injury for children in rural areas [13]. National data comparing rural and urban trauma echoes these other studies, demonstrating increasing mortality with increasing rurality, though homicide rates tend to be higher in urban areas [16]. Across the nation this difference is generally attributed to a lack of pre-hospital care and long transport times in rural areas. However, our data may differ, as both centers in this study are well-established multi-specialty pediatric trauma programs with emergency transport systems.

We found that non-accidental trauma occurred at nearly twice the rate in rural setting when compared to the urban setting. Other studies focusing on non-accidental trauma report the opposite; a greater incidence in urban locations [9], specifically demonstrating more violent pediatric deaths and firearm injuries [13, 17]. These findings; however, were demonstrated in older age groups than our study population. In our cohort, ages 14 and under, all the firearm-related injuries were considered accidental. Consistent with national data [11, 12, 14], the non-accidental trauma group in our study tended to be younger than the general population of pediatric trauma patients.

For nearly half of all non-accidental trauma patients, the primary payer was a government insurance program, this being even more pronounced in the urban setting. Bogumil et al. reported 64% of patients who were victims of non-accidental trauma across the United States had government insurance [15]. Interestingly, in our cohort, despite a higher non-accidental trauma rate, a lower percentage of patients at the rural center were covered by government insurance. In an analysis of injury-related emergency department visits in 14 states including Minnesota it was noted that children from low-income communities were more likely to have injury-related emergency department visits than children from higher-income communities. In the Minnesota cohort of this study, 7% of children admitted with injury were uninsured, 20.9% had Medicaid or government insurance, and 70.8% had private insurance [7]. Despite a higher non-accidental trauma rate, a lower percentage of patients at the rural center were covered by government insurance. Perhaps the rural cohort more accurately reflects the Minnesota population.

In our cohort, patients that were younger, treated at the rural location, or receiving government insurance were at higher risk of non-accidental trauma; however, only age remained an independent predictor on multivariable analysis. Other studies have shown this similar relationship between age and risk for non-accidental trauma. From 2014 reports from the US Department of Health and Human Services, 27.4% of children who suffered non-accidental trauma were younger than 3 years old, comprising 70.7% of child fatalities.

Most children who die of violence-related injuries have had previous treatment for non-accidental injuries [16–18]. Clinicians have been increasingly called upon to expand their role by recognizing non-accidental injuries, intervening, and educating about prevention of such injuries. This can be difficult in emergency settings where time constraints are limiting and providers are often unfamiliar with the patient [19]. Without intervention, children returning to abusive homes face a 44% risk of repeated injury [20].

## Limitations

Our data represents patients from two Midwest level I pediatric trauma centers. This population may not represent those in other areas of the country. Even with the combination of two centers’ data, our sample size was relatively small. Our sample did not include burn injuries (another common cause of non-accidental injury in children) or children who died in the pre-hospital setting. As in other retrospective studies, data used in the analysis are limited to that which is available in the electronic medical record. It is possible that some instances of non-accidental trauma were missed during the patient’s admission. Injury pattern, severity, and patient demographics need to be studied further, to optimize potential preventative strategies. A higher proportion of both urban and rural patients with government insurance are victims of non-accidental trauma than their commercial insurance counterparts. Additional large, multicenter studies are needed to focus on non-accidental trauma in the pediatric trauma population. Outreach efforts therefore should focus on this cohort for educational interventions aimed at reducing childhood trauma in this cohort.

## Abbreviations

ISS: injury severity score
LOS: length of (hospital) stay
SD: standard deviation
y: years

## References

[1] Han PP, Holbrook TL, Sise MJ, Sack DI, Sise CB, Hoyt DB (2011). Postinjury depression is a serious complication in adolescents after major trauma: injury severity and injury-event factors predict depression and long-term quality of life deficits. J Trauma.

[2] Kaske S, Lefering R, Trentzsch H, Driessen A, Bouillon B, Maegele M (2014). Quality of life two years after severe trauma: a single centre evaluation. Injury.

[3] Valadka S, Poenaru D, Dueck A (2000). Long-term disability after trauma in children. J Pediatr Surg.

[4] Discala C, Sege R, Li G, Reece RM (2000). Child abuse and unintentional injuries. J Am Med Assoc.

[5] Holbrook TL, Hoyt DB, Coimbra R, Potenza B, Sise MJ, Sack DI, et al. Trauma in adolescents causes long-term marked deficits in quality of life: adolescent children do not recover preinjury quality of life or function up to two years postinjury compared to national norms. J Trauma 2007;62:577–83; discussion 583. 10.1097/ta.0b013e318031aa97. 10.1097/TA.0b013e318031aa97 17414331

[6] Danseco ER, Miller TR, Spicer RS (2000). Incidence and costs of 1987-1994 childhood injuries: demographic breakdowns. Pediatrics.

[7] Owens PL, Zodet MW, Berdahl T, Dougherty D, McCormick MC, Simpson LA (2008). Annual report on health care for children and youth in the United States: focus on injury-related emergency department utilization and expenditures. Ambul Pediatr.

[8] Coben JH, Tiesman HM, Bossarte RM, Furbee PM (2009). Rural-urban differences in injury hospitalizations in the U.S., 2004. Am J Prev Med.

[9] Mihalicz D, Phillips L, Bratu I (2010). Urban vs rural pediatric trauma in Alberta: where can we focus on prevention?. J Pediatr Surg.

[10] Avdimiretz N, Phillips L, Bratu I (2012). Focus on pediatric intentional trauma. J Trauma Acute Care Surg.

[11] Estroff JM, Foglia RP, Fuchs JR (2015). A comparison of accidental and nonaccidental trauma: it is worse than you think. J Emerg Med.

[12] Carter KW, Moulton SL (2016). Pediatric abdominal injury patterns caused by “falls”: a comparison between nonaccidental and accidental trauma. J Pediatr Surg.

[13] Davies FC, Coats TJ, Fisher R, Lawrence T, Lecky FE (2015). A profile of suspected child abuse as a subgroup of major trauma patients. Emerg Med J.

[14] Litz CN, Ciesla DJ, Danielson PD, Chandler NM (2017). A closer look at non-accidental trauma: caregiver assault compared to non-caregiver assault. J Pediatr Surg.

[15] Bogumil DDA, Demeter NE, Kay Imagawa K, Upperman JS, Burke RV (2017). Prevalence of nonaccidental trauma among children at American College of Surgeons–verified pediatric trauma centers. J Trauma Acute Care Surg.

[16] Deans KJ, Thackeray J, Groner JI, Cooper JN, Minneci PC (2014). Risk factors for recurrent injuries in victims of suspected non-accidental trauma: a retrospective cohort study. BMC Pediatr.

[17] DiScala C, Sege R (2004). Outcomes in children and young adults who are hospitalized for firearms-related injuries. Pediatrics.

[18] King WK, Kiesel EL, Simon HK (2006). Child abuse fatalities: are we missing opportunities for intervention?. Child Abuse Negl.

[19] Tiyyagura G, Gawel M, Koziel JR, Asnes A, Bechtel K (2015). Barriers and facilitators to detecting child abuse and neglect in general emergency departments presented at the eastern society for pediatric research meeting, March 2014, Philadelphia, PA; And the pediatric Academic Societies meeting, May 2014, Vancouve. Ann Emerg Med.

[20] Dakil SR, Sakai C, Lin H, Flores G (2011). Recidivism in the child protection system: identifying children at greatest risk of reabuse among those remaining in the home. Arch Pediatr Adolesc Med.

